# Alphaherpesvirus infection of mice primes PNS neurons to an inflammatory state regulated by TLR2 and type I IFN signaling

**DOI:** 10.1371/journal.ppat.1008087

**Published:** 2019-11-01

**Authors:** Kathlyn Laval, Jolien Van Cleemput, Jonah B. Vernejoul, Lynn W. Enquist

**Affiliations:** Department of Molecular Biology, Princeton University, Princeton, New Jersey, United States of America; University of Florida College of Medicine, UNITED STATES

## Abstract

Pseudorabies virus (PRV), an alphaherpesvirus closely related to Varicella-Zoster virus (VZV) and Herpes simplex type 1 (HSV1) infects mucosa epithelia and the peripheral nervous system (PNS) of its host. We previously demonstrated that PRV infection induces a specific and lethal inflammatory response, contributing to severe neuropathy in mice. So far, the mechanisms that initiate this neuroinflammation remain unknown. Using a mouse footpad inoculation model, we found that PRV infection rapidly and simultaneously induces high G-CSF and IL-6 levels in several mouse tissues, including the footpad, PNS and central nervous system (CNS) tissues. Interestingly, this global increase occurred before PRV had replicated in dorsal root ganglia (DRGs) neurons and also was independent of systemic inflammation. These high G-CSF and IL-6 levels were not caused by neutrophil infiltration in PRV infected tissues, as we did not detect any neutrophils. Efficient PRV replication and spread in the footpad was sufficient to activate DRGs to produce cytokines. Finally, by using knockout mice, we demonstrated that TLR2 and IFN type I play crucial roles in modulating the early neuroinflammatory response and clinical outcome of PRV infection in mice. Overall, these results give new insights into the initiation of virus-induced neuroinflammation during herpesvirus infections.

## Author summary

Herpesviruses are major pathogens worldwide. Pseudorabies virus (PRV) is an alphaherpesvirus related to varicella-zoster virus (VZV) and herpes simplex virus type 1 (HSV1). The natural host is the pig, but PRV can infect most mammals. In these non-natural hosts, the virus causes a severe pruritus called the ‘mad itch’. Interestingly, PRV infects the peripheral nervous system (PNS) and induces a specific and lethal inflammatory response in mice, yet little is know about how this neuroinflammatory response is initiated. In this study, we demonstrated for the first time how PNS neurons tightly regulate the inflammatory response during PRV infection and contribute to severe clinical outcome in mice. Our work provides new insights into the process of alphaherpesvirus-induced neuropathies, leading to the development of innovative therapeutic strategies.

## Introduction

Pseudorabies virus (PRV) is a swine alphaherpesvirus, which infects mucosal epithelia and the peripheral nervous system (PNS) of its host. The virus is closely related to human pathogens herpes simplex virus 1 (HSV1) and varicella-zoster virus (VZV) [1]. In adult swine, wild-type PRV infection causes respiratory and reproductive disorders with a low mortality rate [2]. Infection of neonatal swine, by contrast, is usually fatal resulting from encephalitis [3]. PRV can also infect a wide range of mammals, including dogs and rodents, except higher-order primates [4, 5]. In these non-natural hosts, wild-type PRV infection causes a severe pruritus called ‘the mad itch’ with peracute death [6, 7]. Using a footpad inoculation model, we previously demonstrated that infection with a virulent PRV strain (PRV-Becker), but not with an attenuated live vaccine strain (PRV-Bartha), induces a systemic and lethal inflammatory response in mice [8]. High levels of interleukin 6 (IL-6) and granulocyte colony-stimulating factor (G-CSF) were detected in both plasma and tissues of PRV-Becker infected mice at moribund stage (82 hpi). In addition, we found a strong correlation between PRV-Becker gene expression in the footpad and dorsal root ganglia (DRGs) and the production of both pro-inflammatory cytokines at that time. IL-6 and G-CSF are produced by various cells, including immune cells (neutrophils, macrophages, and T lymphocytes), neurons, and endothelial cells. IL-6 has pleiotropic effects on inflammation, immune response and hematopoiesis [9, 10]. G-CSF regulates neutrophil production and exerts neuroprotective effects through different mechanisms by inhibiting anti-apoptosis and stimulating neuronal differentiation [11–13]. To date, the mechanism by which PRV-Becker initiates the production of G-CSF and IL-6 in mice remains unclear.

The host innate immune system is the first line of defense against herpesvirus infections. This early response is initiated by recognition of viral DNA or RNA through pathogen recognition receptors (PRRs), such as Toll-like receptors (TLRs), IFI16, and cGAS sensors [14, 15]. The detection of viral components by PRRs in host cells activates distinct intracellular signaling cascades, leading to the secretion of type I interferon (type I IFN), and pro-inflammatory cytokines. During HSV1 infection, PRR TLR2 is critical to initiate the innate immune response. Indeed, TLR2 has been shown to mediate the induction of pro-inflammatory cytokines in response to HSV1 infection and contributes to encephalitis in infected mice [16]. More precisely, TLR-2 knockout mice (KO) inoculated intraperitonally with HSV1 showed reduced mortality and had significantly lower serum levels of IL-6 compared to the wild-type mice. TLR2 has also been reported to promote the production of cytokines and chemokines in primary microglia after HSV1 infection [17]. TLRs are expressed in nociceptive neurons and play an important role in neuroinflammation [18, 19]. For instance, it was demonstrated that TLR2 contributes to the nerve injury-induced spinal cord glial cell activation and subsequent pain hypersensitivity [20]. Still, it is not known whether TLR2 signaling is required to regulate the production of IL-6 and G-CSF and to directly contribute to the clinical outcome of PRV infection in mice.

In addition to TLR activity, the IFN response is a critical part of the host innate immune response against viral infections. Type I IFN comprises both IFN-α and -β and is produced as the first wave of antiviral defense [21, 22]. After secretion, IFN-α/β binds to the specific receptors, the IFN-α receptor (IFNAR) 1 and IFNAR 2, expressed on the cell surface. IFNAR engagement activates JAK/STAT signaling pathways, inducing expression of an array of IFN-stimulated genes. The products of many of these genes eventually inhibit viral replication. Like HSV1, PRV has evolved strategies to evade the IFN-mediated immune response. For example, virulent PRV-Becker infection suppresses the expression of most IFNβ-stimulated genes in primary rat fibroblasts through inhibition of STAT1 tyrosine phosphorylation [23]. In addition, another study showed that PRV-Becker infection inhibits the IFN response in swine plasmacytoid dendritic cells (pDCs), while the attenuated vaccine strain PRV-Bartha elicits a much more robust type I IFN response in these cells [24]. This difference was attributed to a deletion of the glycoprotein gE/gI gene complex in the PRV Bartha genome. While type I IFN has been shown to exert an antiviral effect, its role in regulating the production of other pro-inflammatory cytokines and thus controlling PRV-induced neuroinflammation has not been investigated.

Here, we sought to characterize the very early events of the specific inflammatory response induced by PRV infection of mice. We hypothesized that PRV-Becker infection of DRGs that innervate the site of infection, activates the production of G-CSF and IL-6 at very early times post-infection through TLR2 signaling. Because infection by PRV-Becker suppressed type I IFN production, we also determined whether this lack of type I IFN affects the production of cytokines and PRV-induced neuroinflammation in mice. In this study, we first established and leads to the kinetics of G-CSF, IL-6 and type I IFN expression in tissues of PRV-infected and control animals. In addition, we used IFNAR and TLR2 knock-out (KO) mice to dissect the inflammatory response to PRV infection.

## Results

### Virulent PRV-Becker infection induces high levels of IL-6 and G-CSF in mouse tissues early after infection

**Fig 1:**
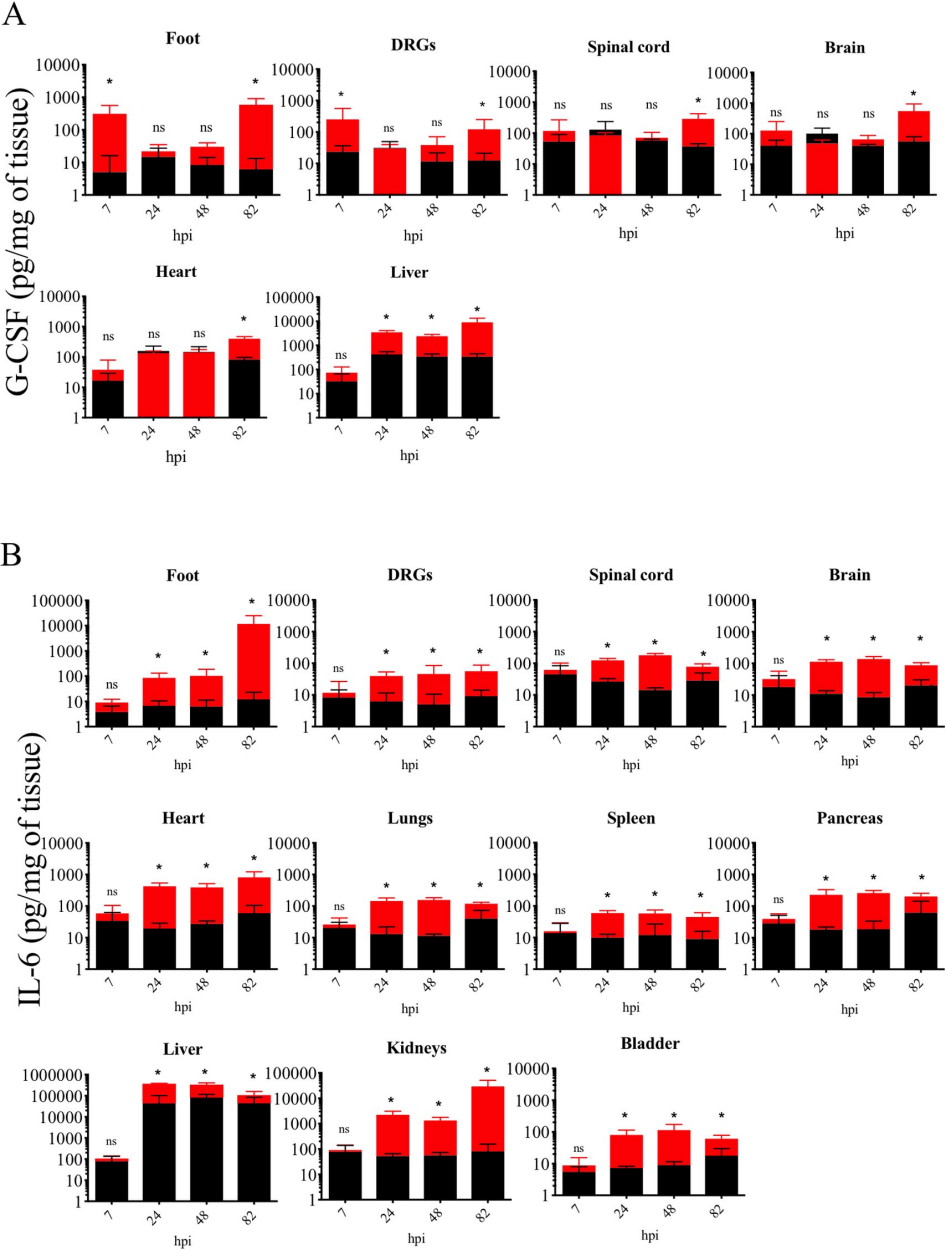
Kinetics of G-CSF and IL-6 production in PRV-Becker infected and control mouse tissues. (A) G-CSF and (B) IL-6 protein levels detected in PRV-Becker infected (red) and control (black) mouse tissues throughout the course of the experiment. Protein levels were quantified by ELISA and expressed as picogram (pg) per milligram (mg) of tissue. Three independent experiments were performed. Five mice per group were used per experiment. *, *P*< 0.05; ns, not significant.

We first determined the early kinetics of G-CSF and IL-6 production in several tissues of PRV-Becker infected mice. As shown in Fig 1A, G-CSF levels were significantly increased in the footpad and DRGs compared to controls as early as 7 hpi (*p*<0.05). By 24 and 48 hpi, G-CSF levels decreased in both tissues and were comparable to controls. At 82 hpi, G-CSF levels increased again in both footpad and DRGs of PRV-Becker infected mice to similar levels as seen at 7 hpi. Significant G-CSF levels were also observed at 82 hpi in spinal cord, brain, heart and liver of PRV-Becker infected compared to control mice. In addition, by 24 hpi, the level of IL-6 was significantly higher in all tissues of PRV-Becker infected mice compared to controls (Fig 1B). For each tissue, the IL-6 concentrations reached a plateau starting from 24 hpi to 82hpi, except for the footpad, which showed increased levels at 82 hpi. Taken together, we conclude that PRV footpad infection induces high G-CSF and IL-6 concentrations in many tissues very early after infection.

### High G-CSF and IL-6 concentrations detected in the footpad and DRGs early upon infection are not caused by immune cell infiltration

To rule out the possibility that the rapid increase of G-CSF and IL-6 in the footpad and DRGs of PRV-Becker infected mice could be attributed to the infiltration of immune cells attracted to the site of infection, sections of both tissues were stained with hematoxylin and eosin (H&E) and compared to control samples.

**Fig 2:**
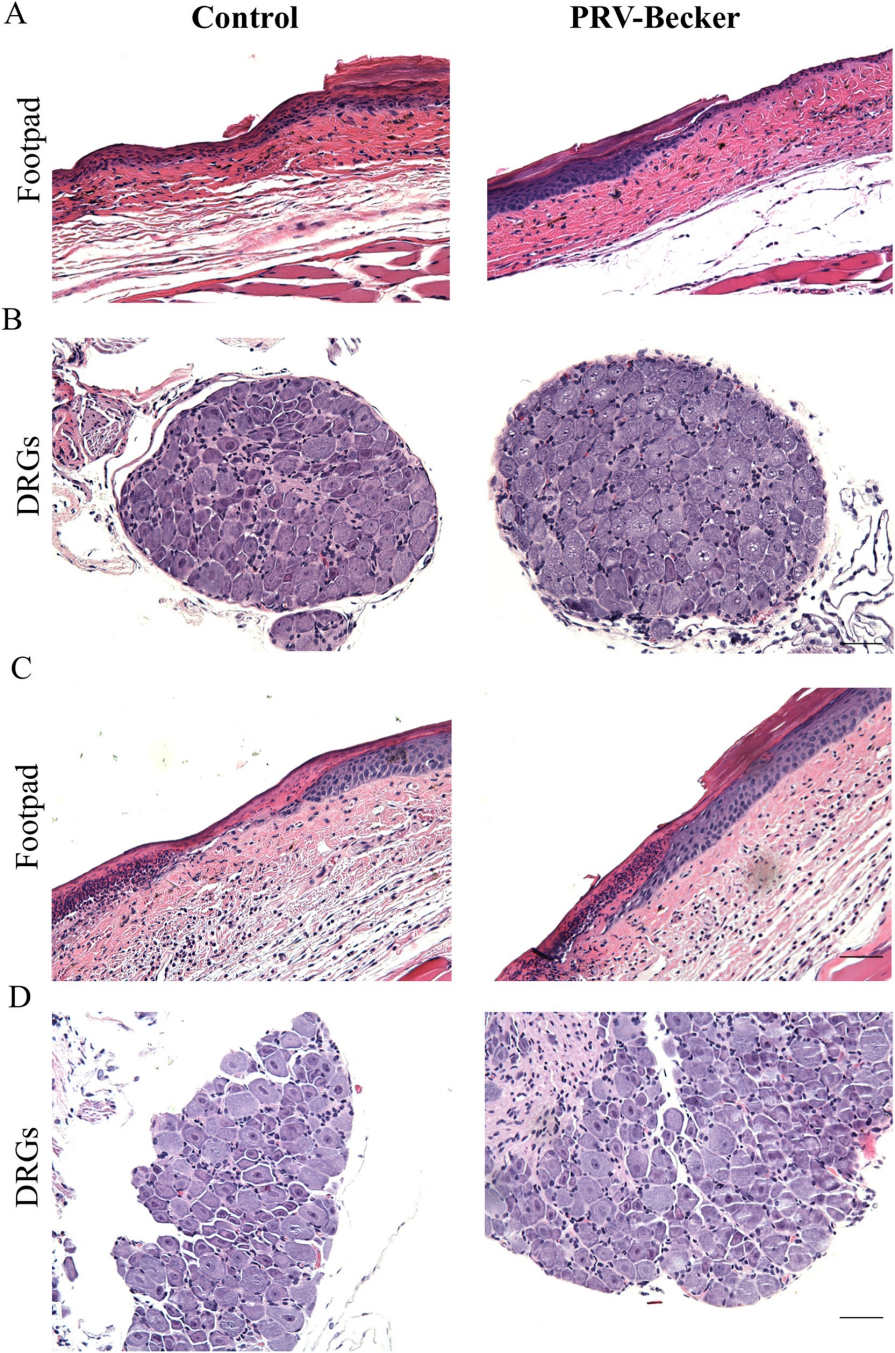
Histopathological findings in footpad and DRGs of PRV-Becker and mock inoculated mice at 7 and 24 h after footpad inoculation. Hematoxylin and eosin (H&E) staining of mouse inoculated footpads and ipsilateral DRGs from control and PRV-Becker inoculated mice at 7 (A and B) and 24 hpi (C and D). Both abraded footpads of PRV-Becker and control mice resulted in minimal to moderate epidermal necrosis in all animals at both time points. No neutrophil infiltrates were observed in DRGs of experimental and control groups. Results are representative of three biological replicates for a given type of tissue. Scale bar (50μm) are indicated for each picture.

Abraded footpads of both PRV-Becker and control mice resulted in minimal to moderate epidermal necrosis in all animals at 7 and 24 hpi, respectively (Fig 2A and 2C). Epidermal necrosis was characterized as segmental loss of the epidermis, sometimes flanked by epidermal thinning and limited neutrophilic accumulation. No viral inclusion bodies were observed within the footpad at both time points. The neutrophilic inflammation was considered to be secondary to loss of the epidermal barrier due to the abrasion rather than a primary effect of the virus. More importantly, no viral inclusion bodies or increase of immune cell infiltrates were detected in the ipsilateral DRGs of control and PRV-Becker infected mice at 7 and 24 hpi (Fig 2B and 2D). Based on the histopathological findings, we can conclude that the high levels of G-CSF and IL-6 detected in the footpad and DRGs, so early after PRV infection, are not caused by the rapid increase of immune cell infiltrates.

### Primary replication of PRV-Becker in the footpad is required to activate DRGs to produce G-CSF and IL-6

Here, we determined whether PRV-Becker replication in DRGs is required to initiate the early production of G-CSF and IL-6. We measured PRV DNA in DRGs at different time post-infection by qPCR analysis. No PRV-Becker DNA was detected in DRGs at 7, 24 and 48 hpi. Therefore, high G-CSF and IL-6 levels detected in tissues of PRV-Becker infected mice at 7 and 24 hpi could not be attributed to extensive viral replication in DRGs.

**Fig 3:**
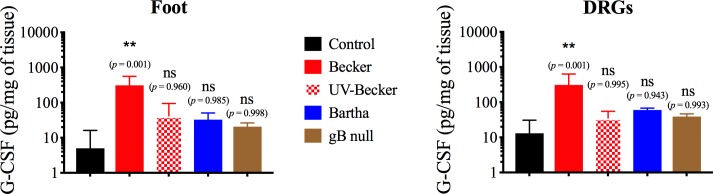
Primary replication of PRV-Becker in the footpad is required to activate DRGs to produce G-CSF at 7 hpi. Mice were inoculated with either mock or PRV-Becker, UV-inactivated PRV-Becker, PRV-Bartha or PRV gB null mutant. At 7 hpi, G-CSF protein levels were measured in the footpad (A) and DRGs (B) of corresponding mice. Protein levels were quantified by ELISA and expressed as picogram (pg) per milligram (mg) of tissue. Three independent experiments were performed. Five mice per group were used per experiment. Data are represented as means + SD. *, *P*< 0.05; ns, not significant compared to control group.

Next, we hypothesized that primary replication of PRV-Becker in the footpad is required to activate DRGs to release these pro-inflammatory cytokines. To test this hypothesis, we compared the levels of G-CSF in the footpad and DRGs of mice either mock inoculated or inoculated with PRV-Becker, UV-inactivated PRV-Becker (UV-Becker), PRV-Bartha, or a gB null mutant (PRV 233) by ELISA at 7 hpi. UV-inactivated PRV-Becker virions deliver their genome to cells, but cannot replicate; PRV-Bartha replicates to a reduced extent compared to PRV-Becker, and PRV 233 can initiate one round of replication in cells but cannot spread further. We found that both mock-inoculated and UV-Becker-inoculated mice showed comparable levels of G-CSF in the footpad and DRGs (Fig 3A and 3B). Unfortunately, we were not able to directly detect viral DNA loads by qPCR because it was not possible to distinguish viral DNA loads between the inoculum and progeny virions at 7 hpi. Also, we could not consistently detect PRV plaques in the mouse footpad by plaque assay so early post-infection. This result clearly indicates that active viral replication in the footpad is required to activate DRGs to produce G-CSF at 7 hpi. Interestingly, mice inoculated with PRV-Bartha or PRV 233 did not show significant G-CSF levels in both footpad and DRGs compared to control groups. Taken together, these results confirm that efficient primary replication and spread of PRV-Becker in the footpad is required to initiate the production of cytokines by DRGs.

### TLR2 facilitates PRV spread from the footpad to the DRG neurons and mediates the neuroinflammatory response in mice

**Fig 4:**
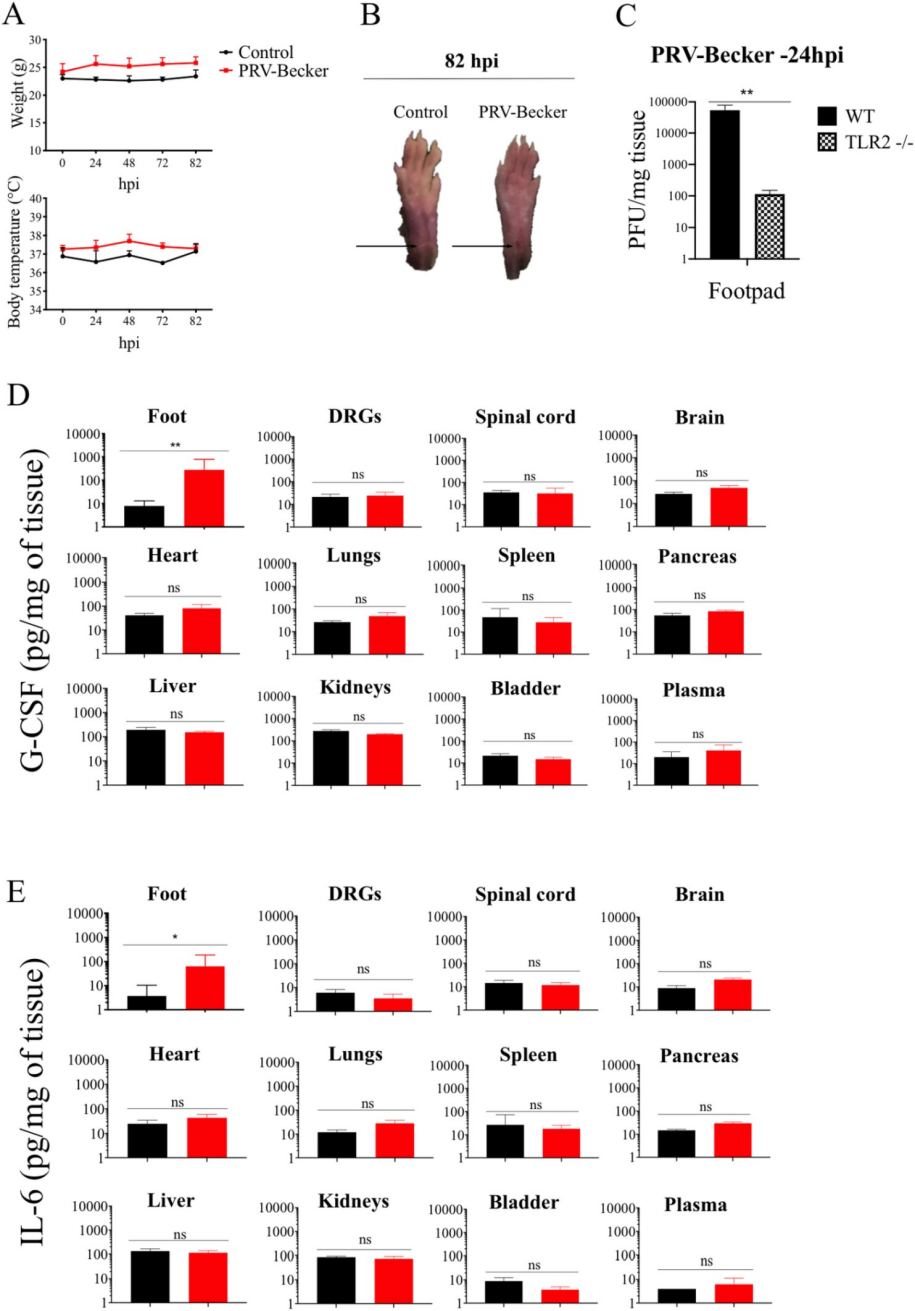
PRV-Becker neither replicates in DRGs nor induces a neuroinflammatory response in TLR2 KO mice. (A) Body weight and temperature of TLR2 KO mice following PRV infection with Becker strain (8.10^6^ PFU) (red) or control (black). (B) Representative images of mouse right hind paws at 82 hpi. Black arrows indicate the site of abrasion. (C) At 24 hpi, PRV DNA was quantitated in mouse footpad by qPCR using UL54 primers. PRV-Becker loads are expressed as plaque forming units (PFU) per mg of tissue. Footpads of PRV-Becker infected WT mice were included as a control. (D) G-CSF and (E) IL-6 protein levels detected in PRV-Becker infected and control mouse tissues at 82 hpi. Protein levels were quantified by ELISA and expressed as pictogram (pg) per milligram (mg) of tissue. (*n* = 5 per group). Two independent experiments were performed. Five mice per group were used per experiment. *, *P*< 0.05; ** *P*< 0.01; ns, not significant.

We investigated whether PRV infection activates DRGs to produce G-CSF and IL-6 through TLR2 signaling. To test this, idea, TLR2 KO mice either were mock infected or inoculated with PRV-Becker at a dose of 8.10^6^ PFU in the footpad and monitored daily for 82 h. Previous work done with WT mice has already been published (8). At the start of the experiment, mice weighed an average of 23 ± 0.7 g and had a mean body temperature of 36.8 ± 0.5°C. No significant increase in body weight and temperature was observed between control and PRV-Becker infected TLR2 KO mice throughout the course of the experiment (Fig 4A). All five PRV-Becker infected mice remained asymptomatic at 82 hpi. The inoculated footpad did not show any signs of inflammation and looked comparable to control footpad (Fig 4B).

We first quantified the PRV load in the footpad of PRV-Becker infected TLR2 KO mice at 24 hpi by qPCR analysis. Footpads of PRV-Becker infected wild-type (WT) mice were used as a control. As shown in Fig 4C, PRV-Becker infected TLR2 KO mice showed significantly less PRV DNA in the footpad (approx. 1.2 x 10^2^ PFU/mg of tissue) compared to PRV-Becker infected WT animals (approx. 5.4 x 10^4^ PFU/mg of tissue). At 82 hpi, we could not detect PRV DNA in the footpad, DRGs, spinal cord and brain of all five PRV-Becker inoculated TLR2 KO mice. Next, we measured the levels of G-CSF and IL-6 in tissues of PRV-Becker inoculated and compared to those in control TLR2 KO mice at 82 hpi. Interestingly, we found that G-CSF and IL-6 levels were only significantly increased in the footpad of PRV-Becker infected TLR2 KO mice compared to control KO group (*p*<0.05) (Fig 4C and 4D). Here, we can conclude that PRV-Becker replication is limited in the footpad of TLR2 KO mice and the infection does not spread and replicate in the DRGs. Similarly, the PRV-induced inflammatory response is restricted to the area of infection, the footpad, as the virus does not activate DRG neurons to produce G-CSF and IL-6 in the absence of TLR2. Overall, these results suggest that TLR2 might be an important receptor for PRV on DRG neurons to facilitate viral spread and to activate PRV-induced inflammation in mice.

### Type I IFN controls both antiviral and neuroinflammatory responses during PRV infection in mice

**Fig 5:**
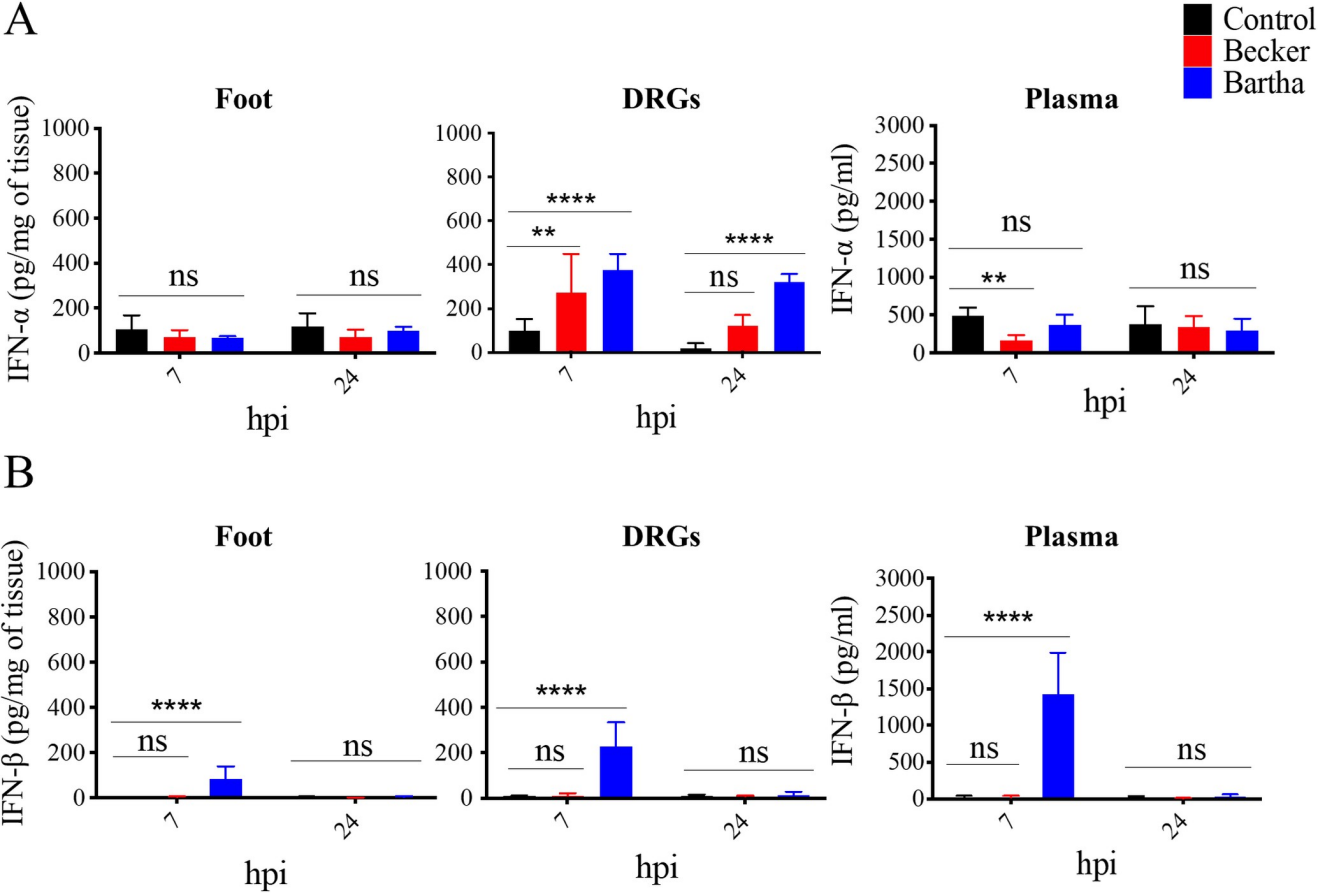
Comparison of type I IFN levels between PRV-Becker and PRV-Bartha infected mice at early time post-infection. (A) IFN-α and (B) IFN-β protein levels detected in the foot, DRGs and plasma of control (black), PRV-Becker (red) and PRV-Bartha (blue) infected WT mice at 7 and 24 hpi. Protein levels were quantified by ELISA and expressed as picogram (pg) per milligram (mg) of tissue. Three independent experiments were performed. Five mice per group were used per experiment. ** *P*< 0.01; **** *P*< 0.0001; ns, not significant.

To investigate the role of type I IFN in controlling the inflammatory response to PRV infection, we first measured and compared the concentrations of IFN-α and IFN-β in the footpad, DRGs and plasma of PRV-Becker, PRV-Bartha infected, and control mice at 7 and 24 hpi. At 7 hpi, both PRV-Becker and PRV-Bartha infections trigger significant IFN-α production in DRGs compared to the control group (*p*<0.01) (Fig 5A). At 24 hpi, IFN-α levels significantly decreased in DRGs of PRV-Becker infected animals while the production remained significantly elevated in DRGs of PRV-Bartha infected mice (*p*<0.0001). No significant IFN-α production was detected in the footpad and plasma of PRV-Becker and Bartha inoculated mice at anytime. Interestingly, PRV-Bartha, but not PRV-Becker, elicited a significant increase of IFN-β in the footpad, DRGs and plasma of inoculated mice at 7 hpi (*p*<0.0001) (Fig 5B). At 24 hpi, IFN-β levels rapidly decreased to control levels.

**Fig 6:**
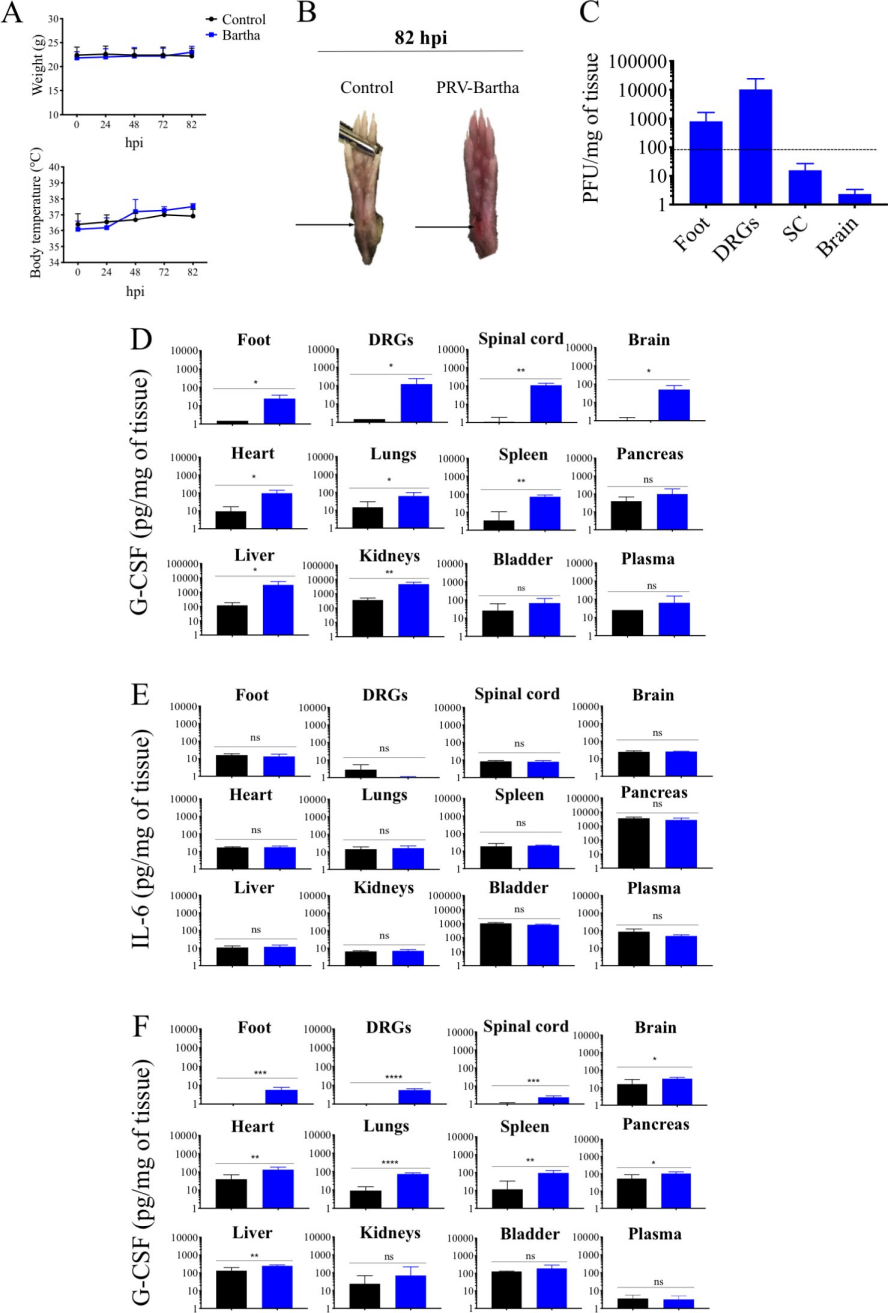
PRV-Bartha replicates efficiently in DRGs and partially induces a neuroinflammatory response in IFNAR KO mice. (A) Body weight and temperature of IFNAR KO mice following PRV infection with Bartha strain (10^8^ PFU) (blue) or control (black). (B) Representative images of mouse right hind paws at 82 hpi. Black arrows indicate the site of abrasion. (C) PRV DNA was quantitated in mouse tissues by qPCR using UL54 primers. PRV-Bartha loads are expressed as plaque forming units (PFU) per mg of tissue. (D) G-CSF and (E) IL-6 protein levels detected in PRV-Bartha infected and control mouse tissues at 82 hpi. (F) G-CSF protein levels detected in PRV-Bartha infected and control mouse tissues at 7 hpi. Protein levels were quantified by ELISA and expressed as pictogram (pg) per milligram (mg) of tissue. Two independent experiments were performed. Five mice per group were used per experiment.*, *P*< 0.05; ** *P*< 0.01; *** *P*< 0.001; ****P< 0.0001; ns, not significant.

Next, we determined if type I IFN plays a role in preventing PRV-Becker-induced disease at late stages of infection using IFNAR KO mice. The footpads of IFNAR KO mice were inoculated with PRV-Bartha at a dose of 10^8^ PFU and monitored daily over an 82 hours period. Previous work done with WT mice has already been published (8). Our expectation was that in the absence of an IFN response, PRV-Bartha infected IFNAR KO mice should develop PRV-Becker-like symptoms. As shown in Fig 6A, PRV-Bartha infected IFNAR KO mice did not show a significant increase of body temperature or weight loss compared to control IFNAR KO animals throughout the course of the experiment. However, PRV-Bartha infected mice did show mild inflammation and redness of the inoculated footpad compared to control group at 82 hpi (Fig 6B). No tremors or intense scratching and biting of the footpad were reported. As shown in Fig 6C, PRV-Bartha DNA was detected only in the footpad (approximately 8 x10^2^ PFU/mg of tissue) and DRGs (approximately 10^4^ PFU/mg of tissue) of IFNAR KO mice. In addition, we quantified the levels of IL-6 and G-CSF in plasma and several tissues of PRV-Bartha infected IFNAR KO mice at 82 hpi by ELISA. G-CSF levels were significantly increased in all tissues, except bladder, of infected mice compared to controls (*p*<0.05) (Fig 6D). However, no significant difference in plasma levels was observed between experimental and control groups. No significant IL-6 increase was detected in the plasma and tissues of PRV-Bartha infected IFNAR KO mice (Fig 6E). Interestingly, high levels of G-CSF were already detected at 7 hpi in several tissues, including PNS and CNS tissues of PRV-Bartha infected mice compared to controls (*p*<0.05) (Fig 6F). Taken together, the data suggest that infection by PRV-Bartha, but not by PRV-Becker, strongly induces production of IFN-β in the footpad, in DRGs, and in plasma of inoculated mice at 7 hpi. In addition to its role in limiting viral replication, our data suggest that type I IFN plays a key role in modulating the early neuroinflammatory response and clinical outcome of PRV infection in mice.

## Discussion

Virulent PRV infection of mice has been shown to induce a specific and lethal systemic inflammatory response [8]. However, the molecular mechanisms by which PRV initiates this inflammatory response remain unknown. Here, using the virulent PRV strain Becker and an attenuated vaccine strain, PRV Bartha, we provide evidence that PRV-Becker infection but not PRV Bartha infection, primes DRG neurons to an inflammatory state at very early time post-infection, which is regulated through TLR2 and type I IFN signaling.

In this study, we first showed that high levels of G-CSF and IL-6, biomarkers for an inflammatory response, are produced in most tissues of PRV-Becker infected mice at early time post-infection. In particular, a peak of G-CSF and IL-6 were detected in the footpad and DRGs at 7 and 24 hpi, respectively. The histopathological examination showed no increase of immune cell infiltrates in both footpad and DRGs at these time points. This result indicates that the early peak of cytokine production detected in both footpad and DRGs of PRV-Becker inoculated animals cannot be attributed to the rapid infiltration of neutrophils to these tissues. We first hypothesized that PRV replication in DRGs was required to initiate this early production. However, we could not detect PRV DNA in the DRGs of PRV-Becker infected animals by RT-PCR at 7 and 24 hpi, respectively. This result is in agreement with a previous study that could only detect PRV in ipsilateral mouse DRGs 48 h after footpad inoculation [5].

Instead, we demonstrated that efficient replication and spread of PRV-Becker in the footpad was required to activate DRGs to produce G-CSF at 7 hpi. Indeed, mice inoculated with the attenuated PRV-Bartha strain or an avirulent PRV strain (PRV 233, gB null mutant) failed to activate cytokine production in DRGs. PRV Bartha is known to have slower replication kinetics than PRV-Becker [25]. Curanović, Lyman [26] demonstrated that PRV-Bartha spreads slower in neuronal circuits due to a mutation in the UL21 gene. In addition, unlike infection by PRV-Becker, infection by PRV-Bartha induces a strong IFN response in cells, which is likely to limit the infection [24]. The PRV gB null mutant can initiate one round of replication but cannot spread from cell to cell [27]. PRV gB protein is a key component of the viral membrane fusion complex, essential for viral entry into neurons [28]. This fusion complex consists of gB/gH/gL that produces fusion pores and enables ions to flow between PNS neurons and cause direct electrical coupling and elevated firing rates *in vitro* [29]. An *in vivo* study demonstrated that PRV-Becker infection of PNS neurons of the submandibular ganglia induces synchronous and cyclical activity in neuronal cell bodies [30]. No signs of synchronous or cyclical firing were observed in PRV gB null infected PNS ganglia. Despite its role in mediating transneuronal spread and electrical coupling in neurons, the involvement of PRV gB in the induction of an early inflammatory response in mice is not known. Here, we speculate that the interaction of PRV gB expressed on infected epidermal cells or new progeny virions with a receptor expressed on DRG neurons is sufficient to activate the early production of G-CSF and IL-6 in DRG neurons and their subsequent release to the footpad through the sciatic nerve via axon terminals.

Interestingly, HSV1 gB has been shown to be a ligand for TLR2 and activates TLR2-dependent NF_k_B signaling *in vitro* [31, 32]. During HSV1 infection, the engagement of TLR2 mediates the production of chemokines, such as CCL2, in infected neurons and facilitates the recruitment of macrophages [33]. In our study, we demonstrated that PRV infection of TLR2 KO mice resulted in the production of IL-6 and G-CSF only in the footpad. All PRV infected KO mice animals remained asymptomatic and survived after 82 hpi. These results are in agreement with a study from Kurt-Jones, Chan [16], which demonstrated that TLR2 mediates the lethal inflammatory cytokine response to HSV1 using KO mice. Similarly, they showed that all HSV1 infected TLR2 KO mice survived 4 days pi compared to 50% infected WT. Even so, no significant difference in HSV1 titer was observed in the brains of TLR2 KO compared to WT mice. Surprisingly, we found that PRV-Becker only replicates in the footpad, but not DRG neurons, of TLR2 KO mice. We speculate that TLR2 might be a receptor required for PRV infection of DRG neurons or that PRV-induced TLR2 activation of DRG neurons triggers the neuroinflammatory response in mice. Future *in vitro* studies will further characterize the interaction of PRV gB with TLR2 expressed in neurons, including a comparison of binding affinities of PRV-Becker and PRV-Bartha gB to TLR2.

Next, we demonstrated that IFN-β, was significantly increased in the foot, DRGs and plasma of PRV-Bartha, but not PRV-Becker, infected mice at 7 hpi. These results are in accordance with a previous study from Lamote, Kestens [24], which showed that PRV-Bartha, but not PRV-Becker, elicits a strong IFN response in pDC. While PRV Bartha infection induces an antiviral state in mice via IFN-β expression, we believe that virulent PRV-Becker infection triggers an exceptional inflammatory response marked by global production of G-CSF and IL6, because it suppresses the IFN response. The imbalance between antiviral and pro-inflammatory immune responses is likely to contribute to the distinct clinical outcomes of PRV-Becker and Bartha infections in mice. In accordance with this finding, we demonstrated that PRV-Bartha infection triggered a modest inflammatory response in mice lacking type I IFN receptor. The inflammatory response was milder than that induced by PRV-Becker infection of WT mice [8].

PRV-Bartha infected IFNAR KO animals only showed inflammation of the inoculated footpad with no signs of morbidity at 82hpi. However, the infection triggered significant G-CSF concentration in tissues compared to KO controls. No significant IL-6 levels were detected in PRV-Bartha infected KO mice. Another interesting finding was that PRV-Bartha replicated to a reduced extent in the footpad and DRGs of KO mice compared to what was seen in PRV-Becker infected WT mice. We think that the limited replication of the attenuated PRV-Bartha in the footpad and DRGs is due in part to the strong IFN-β response, which in turn results in reduced expression of G-CSF in tissues with no subsequent activation of IL-6 production. The absence of high IL-6 concentrations in blood and tissues might explain why the infected animals were still alive at 82 hpi. Indeed, high serum levels of IL-6 have been correlated with disease severity and mortality in cases of bacterial sepsis and viral infections, such as E71 and Sindbis virus infections [34, 35]. IL-6 has also been implicated in the cytokine storm initiated following influenza and severe acute respiratory syndrome (SARS) infections [36, 37]. These results further suggest that PRV-Becker infection in mice induces a biphasic inflammatory response controlled by DRG neurons. In the first phase, PRV-Becker infection of DRG neurons promotes the rapid release of high levels of G-CSF in many uninfected tissues at early time post-inoculation. In the second phase, this high concentration of G-CSF leads to further activation of IL-6 production in uninfected tissues. This expansive expression of G-CSF and IL6 promotes an uncontrolled systemic and lethal inflammatory cytokine response in mice at later time post-inoculation. Overall, our work demonstrates that initial innate immune defenses to infection must be carefully controlled and that virulent infection reflects lack of such control. Indeed, a hallmark of PRV infection is that it is so virulent producing similar lethal symptoms in a broad range of infected mammals (except its natural host). Certainly, evolution of the host-virus interaction is carefully balanced. This assertion is reflected by the fact that PRV infection is lethal for neonatal but not adult swine, which control PRV infection well. Indeed, adult pigs infected with PRV typically exhibit symptoms of respiratory disease with low mortality rate. In contrast, in younger swine, PRV causes an acute neurological disease with a high fatality rate supposedly due to productive viral replication in the CNS [38, 39]. The latter study reported that 2-week-old piglets also exhibited severe neuropathic itch between 3 and 4 days post-infection and piglets were euthanized at 4 days. Moreover, IL-6, IL-10, TNF-α and IFN-γ mRNA were found to be significantly increased in the trigeminal ganglia (TG) of 2-week-old piglets infected with virulent PRV strain NIA3 at 2 days post-infection. The increase in cytokine mRNA expression occurred simultaneously with the appearance of viral mRNA in TG. No clear increase of these cytokines was observed in TG of PRV infected 15-week-old pigs. This study concluded that age-dependent differences in PRV-induced clinical signs are due to enhanced viral replication and associated immunopathology in immature trigeminal ganglion and central nervous system neurons of 2-week old pigs. Based on these results, we do think that the interaction of PRV gB with TLR2 on porcine neurons occurs and thus also triggers a specific inflammatory response in young piglets. In contrast, adult swine infected with PRV do not exhibit neuropathic itch or an aberrant inflammatory response because the infection elicits a strong and protective IFN response in adult animals.

**Fig 7:**
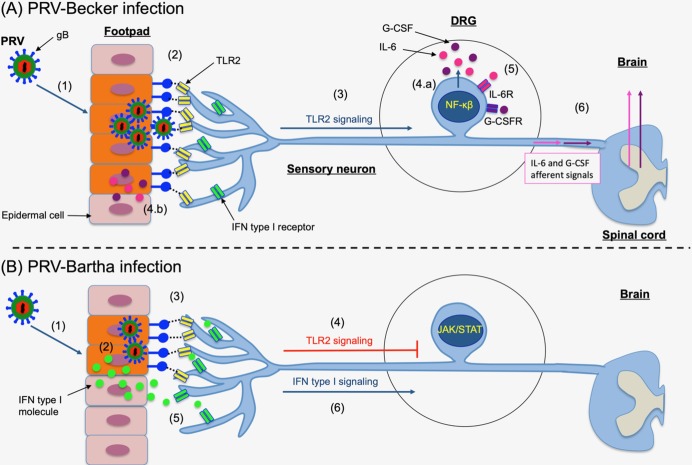
Model of PRV infection and priming of DRG neurons to an inflammatory state versus antiviral state at very early time post-infection. (A) (1) PRV-Becker efficiently replicates and spreads in the epidermal cells of the mouse footpad. (2) PRV gB expressed on infected epidermal cells or on new progeny virions may interact with TLR2 expressed on axonal terminals of DRG neurons that are innervating the footpad. (3) This high level gB-TLR2 interaction activates TLR2 signaling pathway through NF-κβ in DRG neurons and leads to the synthesis of G-CSF and IL-6 molecules. (4) Both cytokines are released in (a) DRG neurons and (b) locally at axon terminals innervating the footpad. (5) G-CSF and IL-6 molecules can directly interact with their receptors (G-CSFR and IL6R) expressed on DRG neurons priming them to an inflammatory state with global effects. (6) G-CSF- and IL-6-mediated signaling pathways may increase the excitability of DRG nociceptors and facilitate synaptic transmission of specific IL-6 and G-CSF afferent signals to the CNS. (B) (1) PRV-Bartha does not replicate and spread efficiently in epidermal cells. (2) The infection induces a rapid type I IFN response in cells. (3) Only a few infected epidermal cells and progeny virions expressing gB on their surfaces can interact with TLR2 found on DRG peripheral axons. (4) Low level gB-TLR2 engagement is below the threshold for activation of TLR2-mediated cytokine signaling pathway. (5) In contrast, type I IFN molecules released from infected epidermal cells bind to their receptors and directly prime DRG neurons to an antiviral state, through activation of type I IFN signaling pathway (JAK/STAT) (6).

Here, we provide a model of PRV infection and priming of DRG neurons to an inflammatory state versus antiviral state at very early time post-infection (Fig 7A and 7B). Virulent PRV-Becker efficiently replicates and spreads in the epidermal cells of the mouse footpad because it suppresses the IFN type I response (Fig 7A). Consistent with the work of others [31, 32], we postulate that PRV gB expressed on infected epidermal cells or on new progeny virions interacts with TLR2 expressed on DRG neurons. This interaction may initiate TLR2 intracellular signaling cascades in these neurons mediating the synthesis of G-CSF and IL-6 in DRGs. A previous study showed that TLR2 signaling inhibits induction of IFN type I by TLR7/9 in murine DCs [40]. This result is consistent with the idea that PRV may have evolved additional mechanisms to suppress the IFN response, not only at the signaling but also induction level. Once released locally, G-CSF and IL-6 molecules can directly interact with their receptors (G-CSFR and IL6R) expressed on DRG neurons priming them to an inflammatory state with global effects [41]. DRG neurons play an important role in the modulation of peripheral and central sensory processing, such as inflammation and neuropathic pain [42–44]. Sensory DRG neurons can also respond to the presence of pathogens, independently of immune cell activation [45]. Interestingly, the interaction of G-CSF with its receptor has been shown to cause hyperexcitability of DRG neurons during colitis [46]. In our model, G-CSF- and IL-6-mediated signaling pathways may increase the excitability of DRG nociceptors and facilitate synaptic transmission to the CNS. We propose that G-CSF and IL-6 specific afferent signals are produced through signaling cascades and then transmitted to the vagus nerve, which innervates visceral tissues. This proposal explains why most tissues collected from PRV-Becker infected mice showed high levels of G-CSF and IL-6 at early time post-infection, independent of a systemic inflammation and evidence of viral infection. Indeed, the nervous system supports homeostasis by modulating the function of organ systems. A well-known example of neural control of inflammation is the inflammatory reflex [47]. This reflex consists of afferent signals that are transmitted in the CNS and culminate in efferent vagus nerve activity that regulates macrophage cytokine release in the spleen [48]. Signals originating in the CNS can travel through the cholinergic vagus nerve and regulate inflammation in peripheral organs innervated by this nerve. This latter hypothesis is supported by a study from Zanos, Silverman [49], which demonstrated that administration of IL-1β and TNF-α to mice activate specific sensory action potentials in the vagus nerve. Each cytokine signal had a specific firing rate and it was suggested that these signals could come from the activation of cytokine specific receptor.

In striking contrast to effects of virulent PRV-Becker infection, we postulate that PRV-Bartha replication and spread is restricted in epidermal cells because infection elicits a rapid type I IFN response (Fig 7B). We suggest that because of this limited replication, only a few infected epidermal cells and progeny virions expressing gB on their surfaces can interact with TLR2 found on DRG peripheral axons. As a result, this low level gB-TLR2 engagement may be below the threshold for activation of TLR2-mediated cytokine signaling pathway. Interestingly, three point mutations have been detected in the functional domain of PRV-Bartha gB fusion protein, which might also affect its efficiency to interact with TLR2 [50]. Another hypothesis is that type I IFN molecules already released from infected epidermal cells can bind to their receptors and directly prime DRG neurons to an antiviral state. In fact, a study from Song, Koyuncu [51] demonstrated that IFN-β applied to isolated axons of cultured PNS neurons induces a local response that limits transport of PRV virus particles. In contrast to G-CSF, type I IFN inhibits excitatory synaptic transmission and nociceptive transmission in the spinal cord [52]. In that scenario, no cytokine afferent signals would be sent to the CNS.

In conclusion, this work reveals some new mechanisms by which PNS neurons control the cytokine response during alphaherpesvirus infection and contribute to severe neuropathies.

## Material and methods

### Viruses

The wild-type virulent PRV strain (PRV-Becker) and live-attenuated PRV strain (PRV-Bartha) were used in this study. PRV-Becker is a virulent field isolate from dog, originally isolated at Iowa State University (USA), with subsequent laboratory passage [53]. PRV-Bartha is a highly passaged vaccine strain, derived from the original Aujeszky strain, which was isolated in Hungary [54]. The attenuated live Bartha strain has mutations in the glycoprotein C (gC), gM, and UL21 genes and a deletion in the unique short region spanning the gI, gE, Us9, and Us2 genes [55–58]. PRV-Becker and PRV-Bartha stocks were grown and titered on monolayers of PK-15 pig kidney cells (ATCC). PRV 232 is a gB-null strain expressing VP26-GFP in a PRV Becker background [27]. The gB-null viral strains were propagated, and their titers were determined in PK15 cells stably transfected with LP64e3, a plasmid expressing PRV gB under the control of the cytomegalovirus (CMV) immediate early promoter. For virus inactivation by UV, a thin layer of viral suspension was exposed to short-wave UV light for 10 min. Absence of viral infectivity was checked by virus titration on PK-15 cells.

### Mice

Male C57BL/6 mice between 5 to 7 weeks old were purchased from The Jackson Laboratory (Bar Harbor, ME). C57BL/6 mice are sensitive to PRV infection, as previously described [59]. Mice deficient for type I IFN α/β receptor (IFNAR) and TLR2 on a C57BL/6 genetic background were also purchased from The Jackson Laboratory (stock number 028288 and 004650, respectively).

### Mouse footpad inoculation model

The protocol used for the footpad inoculation experiments was adapted from the protocol previously described [8]. Briefly, mice were anesthetized with 1–3% isoflurane gas and the right hind footpad, between the heel and walking pads, was gently abraded about 20 times with an emery board until the stratum corneum was removed. A 20-μl droplet of virus inoculum containing 8.10^6^ plaque-forming unit (PFU) of PRV-Becker, UV-PRV-Becker, PRV 232 or 10^8^ PFU of PRV-Bartha, resuspended in medium (Dulbecco modified Eagle medium, 2% fetal calf serum and antibiotics) (Hyclone, GE Healthcare life sciences), was applied onto the abraded area of the skin. Mock-inoculations (medium only) were carried out in parallel. The inoculum was gently rubbed 5 to 10 times with the shaft of an 18-gauge hypodermic needle to facilitate adsorption of the virus. The mice were kept under anesthesia for 30 minutes (min) until the abraded footpad was dry and then the animals were placed in separate cages for further analysis. Mice were weighed daily and temperature measured using a rectal probe. Clinical manifestations of disease were monitored over time as previously described.

### Tissue collection and homogenization

Mice were euthanized by CO_2_ asphyxiation at 7, 24, 48 and 82 hours post-inoculation (hpi). The humane endpoints for PRV-Becker and PRV-Bartha infected WT animals are 82 and 200 hpi, respectively. A set of controls was euthanized at each time point. Fresh tissues (50 to 100 mg), including the footpad, ipsilateral DRGs, spinal cord, brain, heart, lungs, spleen, pancreas, liver, kidneys and bladder were collected, flash-frozen in liquid nitrogen, and stored at -80°C. One hundred milligrams of tissue was weighed and placed in a 2 ml microcentrifuge tube containing a sterile steel bead (Qiagen) and 500 μl modified RIPA buffer containing 0.5M EDTA, pH 8.0; 1M Tris-HCl, pH 8.0; 5M NaCl; 10% SDS and protease cocktail inhibitor tablets (cOmplete Mini EDTA-free, Roche Diagnostics). Tissues were disrupted using a TissueLyser (Qiagen) (20 cycles/s for 2 min, 1 min wait, 20 cycles/s for 2 min) and centrifuged at high speed (17,900 x g) for 10 min. Tissues were stored at -20°C until use in ELISA and qPCR.

### Blood collection

Whole blood (approx. 300 μl) was collected by cardiac puncture after CO_2_ asphyxiation and transferred into 1.5ml EDTA capillary collection tubes (BD Vacutainer). Following collection, samples were first centrifuged for 10 min at 1,500 x g, 4°C to separate cells from plasma and then for 15 min at 2,000 x g, 4°C to deplete platelets. Samples were stored at -80°C until ELISA analysis.

### Histology

At 7 and 24 hpi, footpad and DRGs samples of mock infected or PRV-Becker infected mice were carefully dissected and placed in 10% formalin at 4°C for 24h. The ipsilateral DRGs of lower lumbar and sacral levels were collected for histopathological analyses. Samples were processed and embedded in paraffin and 4–6 μm sections were prepared and stained with hematoxylin and eosin (H&E) by Charles River histopathology services. Three H&E-stained sections per mouse were evaluated for signs of inflammation, epidermal and neuronal necrosis.

### ELISAs

The quantitation of single analyte IL-6, G-CSF, type I IFN (α and β) levels in plasma and/or tissue homogenates were performed using commercial ELISA kits from Thermo Fischer, Qiagen, Biolegend and Invitrogen. The assays were conducted by following the manufacturer's recommendations. All samples were measured in duplicate.

### Quantitative PCR assay (qPCR)

Homogenized tissues were digested with proteinase K (New England Biolabs) in Tween-20 for 60 min at 55°C followed by inactivation for 10 minutes at 95°C prior to qPCR run. Viral genomic DNA was quantified by using UL54 specific primers as previous published [60]. This set of primers (5’-TGC-AGC-TAC-ACC-CTC-GTC-C-3’ and 5’-TCA-AAA-CAG-GTG-GTT-GCA-GTA-AA-3’) (Integrated DNA Technologies) generated a 65 bp fragment of the viral gene UL54 after amplification. Quantitative PCR was performed with Eppendorf Realplex Mastercycler. Reaction mixture was prepared using Kapa Syber Fast qPCR kit and samples were prepared as triplicates. Each experiment was done in duplicates. The amplification reactions were carried out in a total volume of 10 μl, containing 2 μl of template DNA, 5 μl 2X SYBR FAST qPCR Master Mix Universal (Kapa Biosystems), 0.4 μl each of (2.5 μM) forward and reverse primer and 2.2 μl of RNAse free water. The amplification conditions consisted of pre-incubation at 95° for 2 min and 40 cycles of denaturation (5 seconds at 95°C), annealing (20 seconds at 55°C), and extension (10 seconds at 72°C). The quantification cycle (C_t_) was calculated as the cycle number at which the concentration increase became exponential. The specific target amplification was analyzed by melt-curve analysis using the Mastercycler ep realplex 2.2 software.

To quantitate viral DNA, a standard curve was obtained for each experiment by co-amplification of known amounts of PRV DNA. Five consecutive tenfold dilution of PRV stocks was prepared containing from 10^5^ to 10^1^ PFU. PRV-Becker and PRV-Bartha virus stocks (5x10^8^ PFU/ml and 2X10^10^ PFU/ml, respectively) were used as standards to determine how many viral genomes correspond to one plaque forming unit. The amounts of PRV DNA in samples were obtained by plotting C_t_ values onto the standard curve and expressed as PFU/mg of tissue [61]. The conversion of genome copies to PFU was done for convenience of comparing our data to previous published data [8, 61].

### Statistical analyses

Data were pooled from 2–3 independent experiments. Five mice per group were used per experiment. Significant differences (*, P < 0.05; **, P <0.01; ***, P < 0.001; ****, P < 0.0001) between mock-, PRV-Becker- and PRV-Bartha inoculated animals were identified by one way analysis of variances (ANOVA) followed either by a Tukey’s post-hoc test or a two-sided Dunnett’s post-hoc test. If homoscedasticity of the variables was not met as assessed by Levene’s test, the data were log-transformed prior to ANOVA. Normality of the residuals was verified by the use of the Shapiro-Wilk test. If the variables remained heteroscedastic or normality was not met after log-transformation, a Kruskall-Wallis test, followed by a Mann-Whitney post-hoc test were performed. All analyses were conducted in GraphPad Prism v7.0d (Graph Pad Software, La Jolla, CA). Values in the text, graphs, and figure legends throughout the manuscript are means + or ± standard deviations (SDs).

### Ethics statement

All animal experiments were performed in accordance to a protocol (number 2083–16) reviewed and approved by the Institution Animal Care and Use Committee (IACUC) of Princeton University. Princeton personnel are required to adhere to applicable federal, state, local and institutional laws and policies governing animal research, including the Animal Welfare Act and Regulations (AWA); the Public Health Service Policy on Humane Care and Use of Laboratory Animals; the Principles for the Utilization and Care of Vertebrate Animals Used in Testing, Research and Training; and the Health Research Extension Act of 1985.
